# Plasma lipid levels and risk of retinal vascular occlusion: A genetic study using Mendelian randomization

**DOI:** 10.3389/fendo.2022.954453

**Published:** 2022-10-10

**Authors:** Changwei Zheng, Yi Lin, Bingcai Jiang, Xiaomin Zhu, Qianyi Lin, Wangdu Luo, Min Tang, Lin Xie

**Affiliations:** Department of Ophthalmology, The Third Affiliated Hospital of Chongqing Medical University, Chongqing, China

**Keywords:** retinal vascular occlusion, plasma lipid, low-density lipoprotein-cholesterol, high-density lipoprotein-cholesterol, mendelian randomization

## Abstract

The causal effects of plasma lipid levels and the risk of retinal vascular occlusion (RVO) have not been clearly identified, especially for high-density lipoprotein-cholesterol (HDL-C) and low-density lipoprotein-cholesterol (LDL-C). Here, we try to identify these causal risk factors using a two-sample Mendelian randomization (MR) analysis. Single nucleotide polymorphisms (SNPs) were chosen as instrumental variables (IVs). We obtained genetic variants associated with lipid exposure at the genome-wide significance (*P*<5×10^−8^) level from a meta-analysis of GWAS from the Global Lipids Genetics Consortium (GLGC) based on 188,577 individuals of mostly European ancestry for MR analyses. Meanwhile, we used lipid GWAS from UK Biobank (UKB) with a sample size of 115,078 individuals as a supplement. We obtained genetic predictors of RVO from a FinnGen biobank study. We conducted both univariable and multivariable MR (MVMR) analyses to identify the causal effects of RVO. Although inverse variance weighted (IVW) was the primary method used for MR analyses, MR–Egger and weighted-median methods were used as supplements to IVW. We determined the heterogeneity of IVs using Cochrane’s Q test and *I^2^*, and used the MR–Egger intercept and MR-PRESSO Global test to detect horizontal pleiotropy. A leave-one-out sensitivity analysis was conducted by removing a single variant from the analysis. Genetically predicted increased HDL-C level was associated with decreased risk of RVO from GLGC [OR=0.806; 95% CI=(0.659, 0.986); *P*=0.036], which was consistent with UKB results [OR=0.766; 95% CI=(0.635, 0.925); *P*=0.005]. MVMR analysis for plasma lipids [adjusted OR=0.639; 95% CI=(0.411, 0.992); *P*=0.046] or diabetes [adjusted OR=0.81; 95% CI=(0.67, 0.979); *P*=0.029] suggested that low HDL-C may be an independent risk factor for RVO. However, there was no evidence to support a causal association between LDL-C {GLGC [adjusted OR=1.015; 95% CI=(0.408, 2.523); *P*=0.975], UKB [OR=1.115; 95% CI=(0.884, 1.407); *P*=0.359]}, total cholesterol {GLGC [adjusted OR=0.904; 95% CI=(0.307, 2.659); *P*=0.854], UKB [OR=1.047; 95% CI=(0.816, 1.344); *P*=0.716]} or triglycerides {GLGC [OR=1.103; 95% CI=(0.883, 1.378); *P*=0.385], UKB [OR=1.003; 95% CI=(0.827, 1.217); *P*=0.098]} and RVO. Using two-sample MR analysis, our study suggested that dyslipidemia was a risk factor for RVO. Furthermore, our results indicated that a low HDL-C level may be an independent risk factor for RVO, suggesting that controlling HDL-C level may be effective in RVO development.

## Introduction

Retinal vascular occlusion (RVO), including retinal arterial occlusion and retinal vein occlusion, is the second most common retinal vascular disorder and is a major cause of visual impairment (1). Macular edema is the main cause of visual impairment in RVO, while neovascularization of the optic disc and retina is the most serious complication. These complications lead to retinal detachment, vitreous hemorrhage, and neovascular glaucoma, causing irreversible vision loss. Risk factors for RVO are associated with local conditions such as glaucoma (2) and systemic conditions such as hypertension, diabetes, and dyslipidemia (3, 4). Lifestyle factors, including smoking and high body mass index (BMI), are also involved in the development of RVO (4, 5). Due to its multifactorial nature, RVO management remains challenging, such that identifying effective approaches for preventing the development of RVO remains necessary.

A series of clinical studies demonstrated a significant association between plasma lipid levels and the risk of RVO (6–8). However, the association between RVO and LDL-C, HDL-C, and total cholesterol level remains unclear. While some studies have indicated that low HDL-C was an independent risk factor for the development of retinal artery occlusion (9, 10) or retinal vein occlusion (11, 12), other studies failed to show significant correlations (13, 14). Decreased HDL-C even associated with worse visual acuity in retinal artery occlusion (10). Meanwhile, the relationship between LDL-C and RVO remains controversial. An early retrospective study indicated patients with RVO had significantly higher levels of LDL-C (8), and LDL-C levels were independently associated with the occurrence of RVO in multivariate logistic regression analysis (15). But these data are not consistent with previously published results (11). In addition, Song reported total cholesterol was a risk factor for RVO in their meta-analysis (16). Plasma lipid levels are easily altered by medication and lifestyle. These confounding factors may not have been accurately assessed in these studies. These relationship can also be confounded by other unknown factors, often leading to inconsistent and controversial results in traditional retrospective studies (17).

Thus, it is necessary to clarify the causal relationship between RVO and HDL-C, LDL-C and total cholesterol. Mendelian randomization (MR) is an emerging method used for potential causal inference that has demonstrated great success in finding risk factors for diseases. MR treats genetic variations as a natural experiment in which individuals are randomly assigned to higher or lower exposure levels over their lifetime (18). MR is not affected by common confounding factors, and the causal sequence is reasonable (19). To date, the two-sample MR analysis has not been used to examine the effects of plasma lipid levels on the risk of RVO. The ultimate aim of the MR analyses performed in the present study is to clarify the causal relationship between plasma lipids and RVO.

## Methods

### Study design

**Figure 1:**
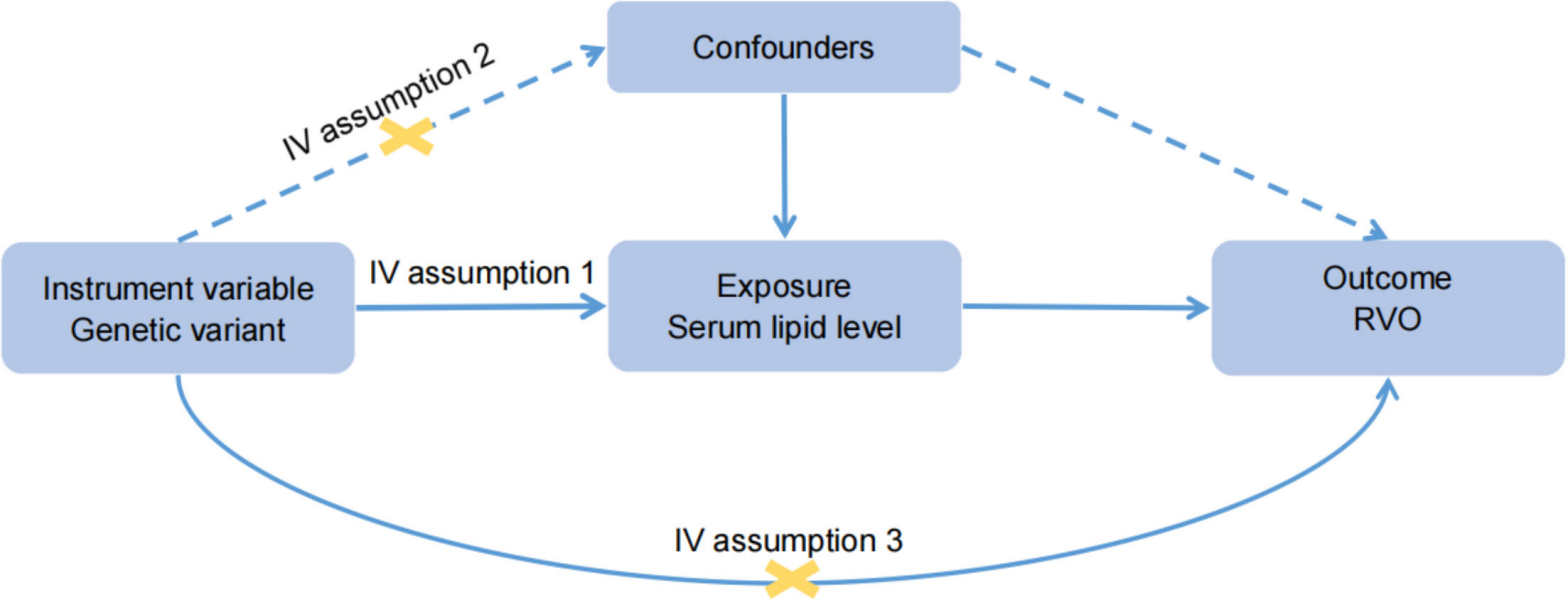
Basic assumptions of Mendelian randomization. Assumption 1: The genetic variants were strongly and causally related to exposure; Assumption 2: the genetic variants were not associated with any potential confounders; Assumption 3: each genetic variant and the outcome did not have common causes.

We followed the Strengthening the Reporting of Observational Studies in Epidemiology using Mendelian Randomization (STROBE-MR) guideline to report the MR study (19). MR analyses were performed to estimate the causal relationships between lipid levels and RVO risk. The MR study was performed using publicly available GWAS summary statistics, and ethical approval was obtained in all original studies. The following assumptions are made for MR inference: 1), The genetic variants were strongly and causally related to exposure; 2), the genetic variants were not associated with any potential confounders; 3), each genetic variant and the outcome did not have common causes (**Figure 1**). In univariable MR analysis, we simply tested the causation between each lipid risk factor and RVO. However, in MVMR analysis, we included the significant risk factors (HDL-C, LDL-C and total cholesterol) from the univariable analysis and tried to identify the independent risk factor for plasma lipids. In addition, a low HDL-C level is associated with a higher risk of type 2 diabetes (20) that increases the risk of RVO. So we also used MVMR to mitigate potential pleiotropic effects *via* diabetes in UK Biobank (UKB).

### Data sources and instrumental variable extraction

Four lipid phenotypes, HDL-C, LDL-C, triglycerides and total cholesterol, were included in this study as the exposure variables. For the exposure data, publicly available summary statistics data, based on 188,577 individuals of mostly European ancestry, were identified through a meta-analysis of GWAS from the Global Lipids Genetics Consortium (GLGC) in 2013 (21). This study was adjusted for sex, age, age squared, body mass index, and genotyping chips. Since the GWAS from GLGC contained mixed populations, we also used the lipid GWAS with a sample size of 115,078 individuals from UKB, conducted by Nightingale Health 2020 as a supplement. For the outcome data, we extracted the summary statistic datasets from a recent GWAS of RVO conducted by the FinnGen biobank adjusting for age, sex, genotyping batch and ten first principal components. Participants included 1,595 cases and 203,108 controls of individuals with European ancestry, and access to the MR-Base database was free (http://www.mrbase.org/). We obtained genetic associations with type 2 diabetes adjusted for BMI from Mahajan et al. (22). We included SNPs reaching GWAS (GWAS *P*<5×10^−8^) whose minor allele frequency was > 0.01. These SNPs were clumped based on the linkage disequilibrium (r^2^<0.001) in the given genome region (kb=10,000). Palindromic SNPs were discarded. To evaluate the strengths of the selected genetic predictors for the RVO, an *F* statistic (*F*=beta^2^/se^2^; beta: beta for the SNP-exposure association, se: variance) was calculated for each SNP (23). Generally, an *F* value >10 indicates no obvious bias is caused by weak IVs. SNPs with less statistical power were removed (*F* value <10). MR-Steiger filtering was used to remove variations that were more strongly correlated with RVO than with lipids (24). Full details of the SNPs, MR-Steiger and *F* value are provided in the **Supplementary Datasets 1**–**8**. We extracted the SNPs having an association with at least one of LDL-C, HDL-C, and total cholesterol at GWAS significance (*P*<5×10^−8^) from GLGC for MVMR analysis. We used the IVW model and excluded triglycerides from this analysis. Since there is no association between triglycerides and RVO, meaning that there is no need to adjust for secondary exposure.

### Statistical analyses

We used a standard inverse variance weighted (IVW) method to estimate the causal effects in the two-sample MR analysis. In addition, we used MR–Egger and weighted-median methods as supplements to IVW. We determined the heterogeneity of IVs using Cochrane’s Q test and *I^2^. P*<0.05 of Cochrane’s Q indicated the existence of heterogeneity (25). *I^2^* was categorized as low, moderate, or high, and a value >25% was regarded as significant heterogeneity (26). In addition, the heterogeneity within the MR–Egger analysis was evaluated by calculating Rucker’s Q (27). *P*<0.05 of Cochran’s Q and Rucker’s Q (Q-Q’) indicates MR-Egger to be a better method because of unbalanced horizontal pleiotropy (27). We used the MR–Egger intercept (28) and MR-PRESSO Global test (29) to detect horizontal pleiotropy, with a *P* value <0.05 indicating that the exposure may have the other pathway for IVs to influence the outcome. We conducted a leave-one-out sensitivity analysis by removing a single variant from the analysis (**Supplementary Figures 1–8**).

*P<*0.05 was considered to be statistically significant. All MR analyses were performed using R software (4.1.2) and the R packages “TwoSampleMR” and “MR-PRESSO”.

## Results

**Figure 2:**
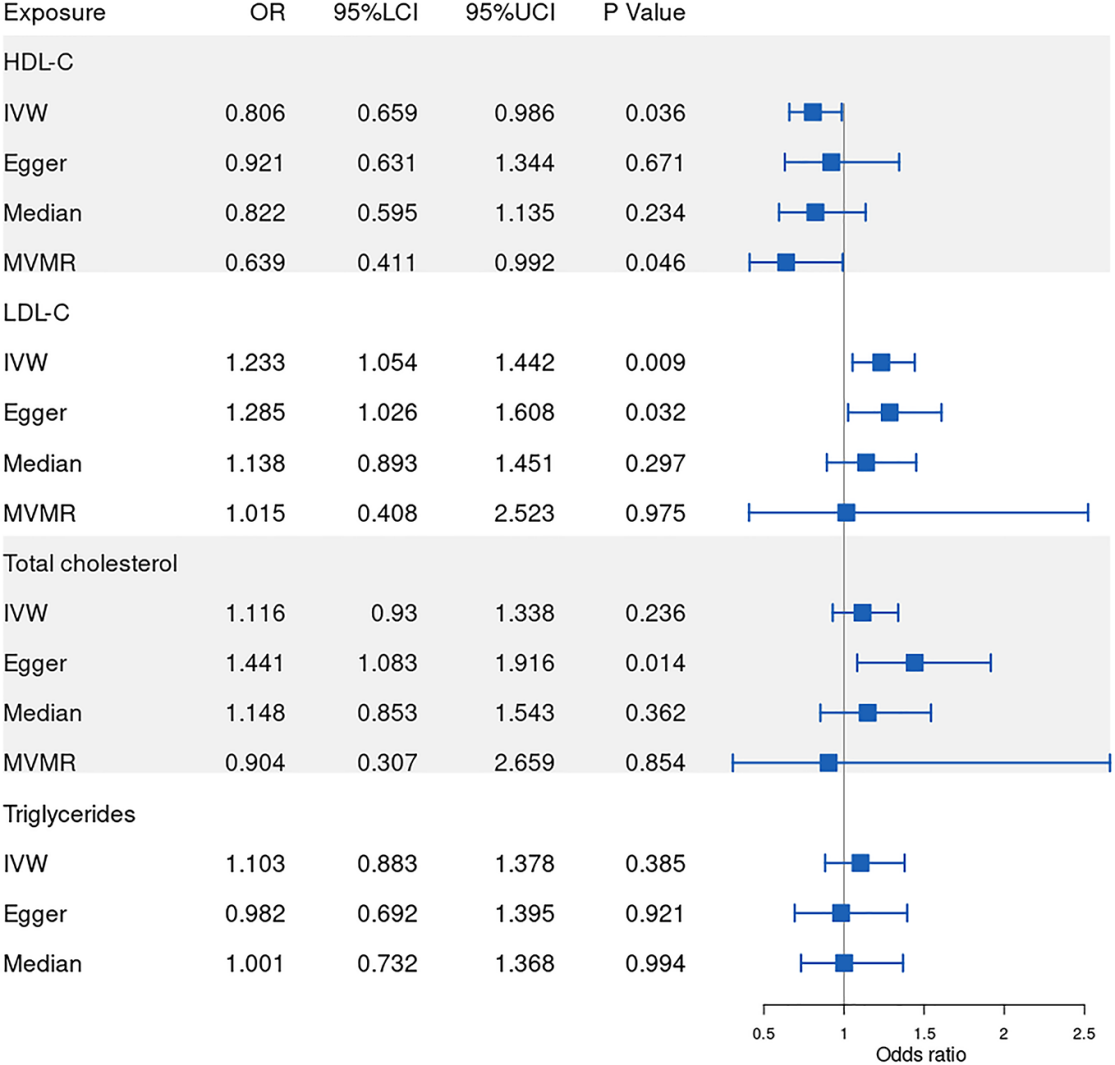
Forest plot of Mendelian randomization results from GLGC. IVW: inverse variance weighted, Egger: MR–Egger, Median: weighted-median, MVMR: multivariable mendelian randomization, 95%LCI: lower limit of 95% CI, 95%UCI: upper limit of 95% CI. GLGC, Global Lipids Genetics Consortium.

**Figure 3:**
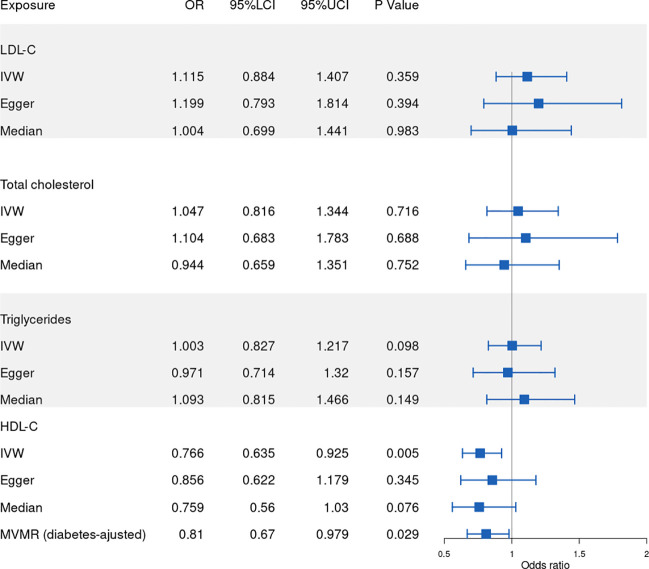
Forest plot of Mendelian randomization results from UKB. IVW: inverse variance weighted, Egger: MR–Egger, Median: weighted-median, 95%LCI: lower limit of 95% CI, 95%UCI: upper limit of 95% CI. UKB: UK Biobank.

Our results show that 86, 77, 53, 81 SNPs from GLGC and 78, 45, 65, 57 SNPs from UKB were associated with HDL-C, LDL-C, triglycerides and total cholesterol respectively (**Supplementary Datasets 1**–**8**). The software tool SNiPA (30) was used to identify the overlapping SNPs between the two independent datasets (SNPs in high LD, r^2^>0.8). There were 54 and 32.93% overlapping SNPs in HDL-C, 34 and 27.87% overlapping SNPs in LDL-C, 20 and 16.95% overlapping SNPs in triglycerides, and 58 and 42.03% overlapping SNPs in total cholesterol. We listed these overlapping SNPs in **Supplementary Table 2**. The *F* statistics were all greater than the empirical threshold of 10, suggesting that all SNPs had sufficient validity, and the minimum *F* statistics in each subgroup are shown in **Table 1**. The explained variances varied from 4.31% to 9.41% (**Table 1**). The results of the MR analysis from GLGC are presented in **Figure 2** and the results from UKB are presented in **Figure 3**.

**Table 1:** Mendelian randomization results of lipid traits on RVO.

					Cochrane's Q statistic		MR-Egger test		MR-PRESSO Global test
	NSNP	R^2^ (%)	F statistic	I^2^ (%)	Q	P value	intercept	P value	P value
GLGC									
HDL-C	86	6.42	29.95	5.82	90.256	0.328	-0.007	0.418	0.345
LDL-C	77	9.41	27.79	0	74.604	0.523	-0.004	0.615	0.509
Triglycerides	53	5.46	29.86	0	45.623	0.721	0.007	0.405	0.738
Total cholesterol	81	8.36	28.93	11.42	90.315	0.202	-0.018	0.018	0.205
UKB									
HDL-C	78	7.42	26.37	3.5	79.793	0.391	-0.008	0.404	0.394
LDL-C	45	4.31	28.35	0	41.444	0.582	-0.005	0.678	0.576
Triglycerides	65	4.45	25.23	0	63.561	0.492	0.003	0.405	0.79
Total cholesterol	57	7.22	27.11	14.17	65.242	0.186	-0.003	0.803	0.176

NSNP, number of single nucleotide polymorphisms; RVO, retinal vascular occlusion; GLGC: Global Lipids Genetics Consortium; UKB, UK Biobank; R^2^, phenotype variance explained by genetics.

### Causal effect of HDL-C on RVO

In our MR analysis of the relationship between HDL-C level and RVO, our overall causal estimate obtained using the IVW method suggested a causal association between them. As shown in **Figure 2**, genetically predicted increased HDL-C level was associated with decreased risk of RVO [OR=0.806; 95% CI=(0.659, 0.986); *P*=0.036)] from GLGC. Similarly, genetically predicted increased HDL-C level was associated with lower risk of RVO [OR=0.766; 95% CI=(0.635, 0.925); *P*=0.005] from UKB (**Figure 3**). Moreover, our MVMR analysis for plasma lipids [adjusted OR=0.639; 95% CI=(0.411, 0.992); *P*=0.046] or diabetes [adjusted OR=0.81; 95% CI=(0.67, 0.979); *P*=0.029] suggested that low HDL-C may be an independent risk factor for RVO. Our sensitivity analysis showed that there were no heterogeneity *via* Cochrane’s Q test [GLGC (Q=90.256, *P*=0.328); UKB (Q=79.793, *P*=0.391)] or *I^2^* [GLGC (*I^2 =^* 5.82%); UKB (*I^2 =^* 3.5%)] and no horizontal pleiotropy *via* the MR–Egger test [GLGC (intercept=-0.007, *P*=0.418); UKB (intercept=-0.008, *P*=0.404)] or MR-PRESSO Global test [GLGC (*P*=0.345); UKB (*P*=0.394)], as shown in **Table 1**. Our leave-one-out analysis found that no single genetic variant strongly drove the overall effect of plasma lipids on RVO (**Supplementary Figure 1**). Using the MR-Steiger test, none of the variants were removed and results remained unchanged.

### Causal effect of LDL-C on RVO

Next, we assessed the causal relationship between LDL-C level and RVO. High LDL-C level was suggestively associated with the risk of RVO using the IVW analysis method [OR=1.233; 95% CI=(1.054, 1.442); *P*=0.009)] (**Figure 2**). However, UKB results showed a null effect on RVO [OR=1.115; 95% CI=(0.884, 1.407); *P*=0.359)]. This was consistent with our MVMR analysis for lipids [adjusted OR=1.015; 95% CI=(0.408, 2.523); *P*=0.975]. Nonsignificant heterogeneity was detected across the instrument SNP effects by Cochrane’s test [GLGC (Q=74.604, *P*=0.523); UKB (Q=41.444, *P*=0.582)] or *I^2^* [GLGC (*I^2 =^* 0%); UKB (*I^2 =^* 0%)] (**Table 1**). The MR–Egger intercept test did not reveal any signs of horizontal pleiotropy regarding LDL-C level [GLGC (intercept=-0.004, *P*=0.615); UKB (intercept=-0.005, *P*=0.678)], similar to results obtained using MR-PRESSO [GLGC (*P*=0.509); UKB (*P*=0.576)] (**Table 1**). The leave-one-out test showed that MR results were not significantly affected by a single SNP leave-out (**Supplementary Figure 2**). No one SNP was excluded by MR-Steiger.

### Causal effect of triglycerides on RVO

We further investigated the relationship between triglycerides and the risk of RVO using MR analysis. The causal association of genetically predicted triglyceride level with RVO determined by the IVW [OR=1.103; 95% CI=(0.883, 1.378); *P*=0.385] demonstrated null effects (**Figure 2**), consistent with results obtained from UKB [OR=1.003; 95% CI=(0.827, 1.217); *P*=0.098]. Cochrane’s test [GLGC (Q=45.623, *P*=0.721); UKB (Q=63.561, *P*=0.492)] or *I^2^* [GLGC (*I^2 =^* 0%); UKB (*I^2 =^* 0%)] detected no significant heterogeneity among SNPs. Subsequently, MR–Egger regression analysis [GLGC (intercept=0.007, *P*=0.405); UKB (intercept=0.003, *P*=0.405)] or MR-PRESSO [GLGC (*P*=0.738); UKB (*P*=0.79)] (**Table 1**) detected no horizontal pleiotropy.

### Causal effect of total cholesterol on RVO

Finally, we explored the causal relationship between total cholesterol and RVO. The MR–Egger test showed a causal effect between total cholesterol level and the risk of RVO [OR=1.441; 95% CI=(1.083, 1.916); *P*=0.014)], while the IVW method showed a null causal effect [OR=1.116; 95% CI=(0.93, 1.338); *P*=0.236]. The MVMR analysis for lipids [adjusted OR=0.904; 95% CI=(0.307, 2.659); *P*=0.854] and UKB results [OR=1.047; 95% CI=(0.816, 1.344); *P*=0.716] demonstrated null effects. No heterogeneity presence by Cochrane’s test [GLGC (Q=90.315, *P*=0.202); UKB (Q=65.242, *P*=0.186)] or *I^2^* [GLGC (*I^2 =^* 11.42%); UKB (*I^2 =^* 14.17%)]. Although pleiotropy was present (MR–Egger intercept =-0.018, *P*=0.028) in GLGC, no significant outlier was tested by MR-PRESSO (*P*=0.205) **(** **Table 1** **).** No pleiotropy was present in UKB [(MR–Egger intercept =-0.003, *P*=0.803); MR-PRESSO (*P*=0.176)]. In addition, Rucker’s Q of total cholesterol was significantly lower (*P*=0.02) than Cochran’s Q, indicating MR-Egger to be a better method because of unbalanced horizontal pleiotropy (**Supplementary Table 1**). So we adopted the results analyzed by MR–Egger in GLGC. No one SNP was excluded by MR-Steiger.

## Discussion

This study substantiates the conclusion that low HDL-C is a risk factor for RVO both from GLGC and UKB. In contrast, we found no evidence of a causal association between LDL-C, total cholesterol or triglycerides and RVO. After adjusting for each lipid profile component or diabetes using MVMR, our results indicated the association between HDL-C and the risk of RVO, suggesting that low HDL-C may be an independent risk factor for RVO.

Although the exact pathogenesis of RVO remains elusive, numerous studies have reported that RVO is associated with atherosclerosis, a chronic inflammatory disease of the arteries (5, 15, 31). Hyperlipidemia is a major risk factor for atherosclerosis, and abnormal lipid metabolism is an important component of atherosclerosis. Meanwhile, LDL-C is the most abundant atherogenic lipoprotein in plasma (32) and leads to the initiation and progressive growth of atherosclerotic plaque increases in a dose-dependent manner (33). Thus, LDL-C may play an important role in the development of RVO. And the association between elevated LDL-C level and the development of RVO has been demonstrated by previous studies (8, 15). GLGC results suggested that high LDL-C may be a risk factor for RVO. However, many lipid trait SNPs carry pleiotropic lipid trait effects and overlap between genetic determinants of LDL-C and HDL-C is widespread (34). MVMR is useful to estimate the direct causal effect of each lipid profile component, independent of the other lipid profile variables (35). MVMR analysis for plasma lipids suggested that high LDL-C may not be an independent risk factor for RVO. This result was consistent with UKB.

A few studies have reported that the total cholesterol is significantly related to the risk of RVO (15, 16). However, MVMR analysis for lipids and UKB results showed null effects on RVO. Total cholesterol and LDL-C are easy direct targets for medical treatments, such that baseline total cholesterol and LDL-C level may vary substantially in clinical trials, complicating the interpretation of results regarding RVO risk. Our findings suggested that total cholesterol and LDL-C may not be independent risk factors for RVO. Thus, more studies are required to determine the causal relationship between total cholesterol, LDL-C and RVO.

Unlike LDL-C, HDL-C is widely believed to exert atheroprotective effects. Despite the evidence from various previous reports indicating an association between HDL-C and the risk of RVO, this conclusion remains controversial. HDL-C directly mediates reverse cholesterol transport that hinders the accumulation of cholesterol in the arterial wall and prevents the progression of atherosclerosis (36). Additionally, HDL-C has been shown to inhibit the endothelial inflammatory response and oxidation of LDL-C (37). Through these underlying mechanisms, increasing HDL-C level may have improved the endothelial health of the retinal vessel, and decreased the risk of RVO. An early Study from Beaver Dam Eye demonstrated a relationship between higher baseline HDL-C levels and RVO, but the results were not statistically significant (38). Recently, two national cohort studies verified that low HDL-C was a risk factor for retinal artery occlusion and retinal vein occlusion (9, 11), consistent with our MR results both from GLGC and UKB. The MR study reported here provides genetic evidence that low HDL-C is a risk factor for RVO. In addition, many lipid trait SNPs carry metabolic syndrome trait effects, especially diabetes, which is an important and concerning confounder. A low HDL-C level is associated with a higher risk of type 2 diabetes (39) and studies have shown that RVO is associated with diabetes (40). After adjusting for diabetes, MVMR analysis demonstrates that low HDL-C may be an independent risk factor for RVO, suggesting that controlling of HDL-C level may be effective in managing RVO.

To the best of our knowledge, this is the first study to clarify a causal associations between plasma lipid levels and RVO risk using the MR method. This study has several strengths. The use of MR design reduces the risk of bias from confounding factors and is suitable for causal inference. This MR study was analyzed using two independent lipid GWAS datasets, which makes the results more reliable. However, our MR study does have several limitations that should not be ignored. The greatest concern in MR studies is the horizontal pleiotropy, which occurs when the genetic variants influence the outcome of more than one pathway (25). Furthermore, the horizontal pleiotropy can be classified into uncorrelated pleiotropy and correlated pleiotropy, in which the former means SNPs affect exposure and outcome independently and the latter means SNPs affect the two traits through a shared pathway (41). In an effort to minimize this bias, we used two main means to detect the uncorrelated pleiotropy, the MR–Egger intercept and MR-PRESSO methods. However, unmeasured confounding may still exist such as correlated pleiotroy. Therefore, MR-Steiger test was used to filter all selected SNPs, hoping to minimize the bias (24). However, it is not possible to completely rule out the presence of residual pleiotropy. Last but not least, care should be taken when expanding our conclusions to other populations, as the present MR analysis utilized subjects of primarily Europeans.

In conclusion, the present study indicated that exposure to abnormal lipid level may increase the risk of RVO. Furthermore, our findings demonstrated a causal association between HDL-C and the risk of RVO, after adjustment for lipid components or diabetes, suggesting that controlling of HDL-C level may be effective in managing RVO.

## Data availability statement

The original contributions presented in the study are included in the article/**Supplementary Material**. Further inquiries can be directed to the corresponding author.

## Author contributions

CZ performed the main data analysis and wrote the draft of the manuscript. YL, BJ, XZ, QL, WL, and MT contributed to the data analysis and manuscript revision. LX supervised the whole research and is responsible for the integrity of data analysis. All authors contributed to the article and approved the submitted version.

## Funding

The research work was supported by the Natural Science Foundation of China (Grant no. 81470629 and 81670860; Chongqing, China), Chongqing Natural Research Foundation (No. cstc 2018jcyjAX0034; Chongqing, China).

## Conflict of interest

The authors declare that the research was conducted in the absence of any commercial or financial relationships that could be construed as a potential conflict of interest.

## Publisher’s note

All claims expressed in this article are solely those of the authors and do not necessarily represent those of their affiliated organizations, or those of the publisher, the editors and the reviewers. Any product that may be evaluated in this article, or claim that may be made by its manufacturer, is not guaranteed or endorsed by the publisher.
