# Supplementary material for: Plasma lipid levels and risk of retinal vascular occlusion: A genetic study using Mendelian randomization
Source: Front Endocrinol (Lausanne). 2022 Oct 10;13:954453. doi: 10.3389/fendo.2022.954453 (PMC9588969; doi:10.3389/fendo.2022.954453)
Supplement: Supplementary file 9 [file Table_1.docx]

Figure 1: Leave-one-out analysis plots for HDL-C on the risk of RVO from GLGC.

Figure 2: Leave-one-out analysis plots for LDL-C on the risk of RVO from GLGC.

Figure 3: Leave-one-out analysis plots for triglycerides on the risk of RVO from GLGC.

Figure 4: Leave-one-out analysis plots for total cholesterol on the risk of RVO from GLGC.

Figure 5: Leave-one-out analysis plots for HDL-C on the risk of RVO from UKB.

Figure 6: Leave-one-out analysis plots for LDL-C on the risk of RVO from UKB.

Figure 7: Leave-one-out analysis plots for triglycerides on the risk of RVO from UKB.

Figure 8: Leave-one-out analysis plots for total cholesterol on the risk of RVO from UKB.

Figure 1：


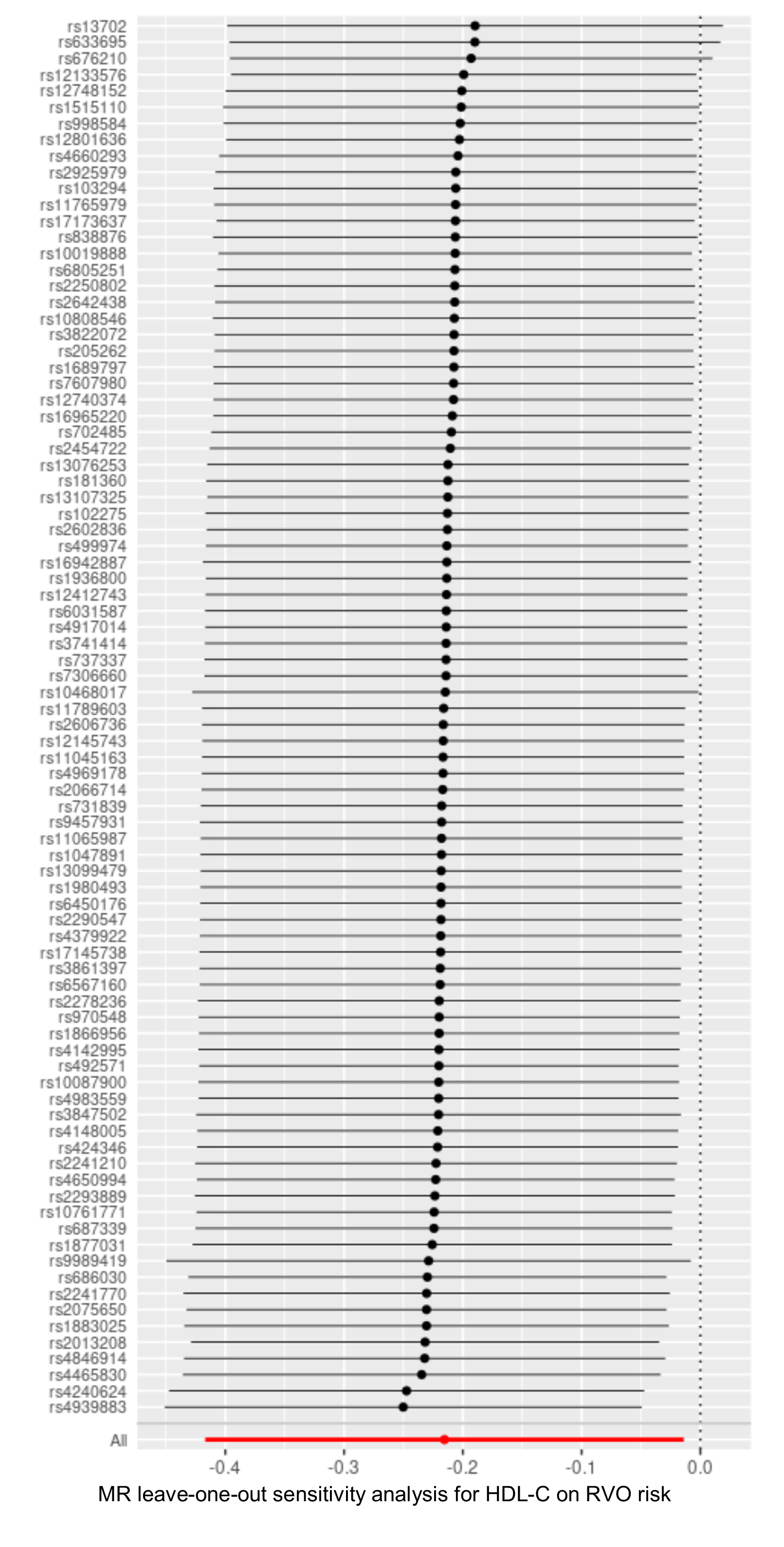


Figure 2：


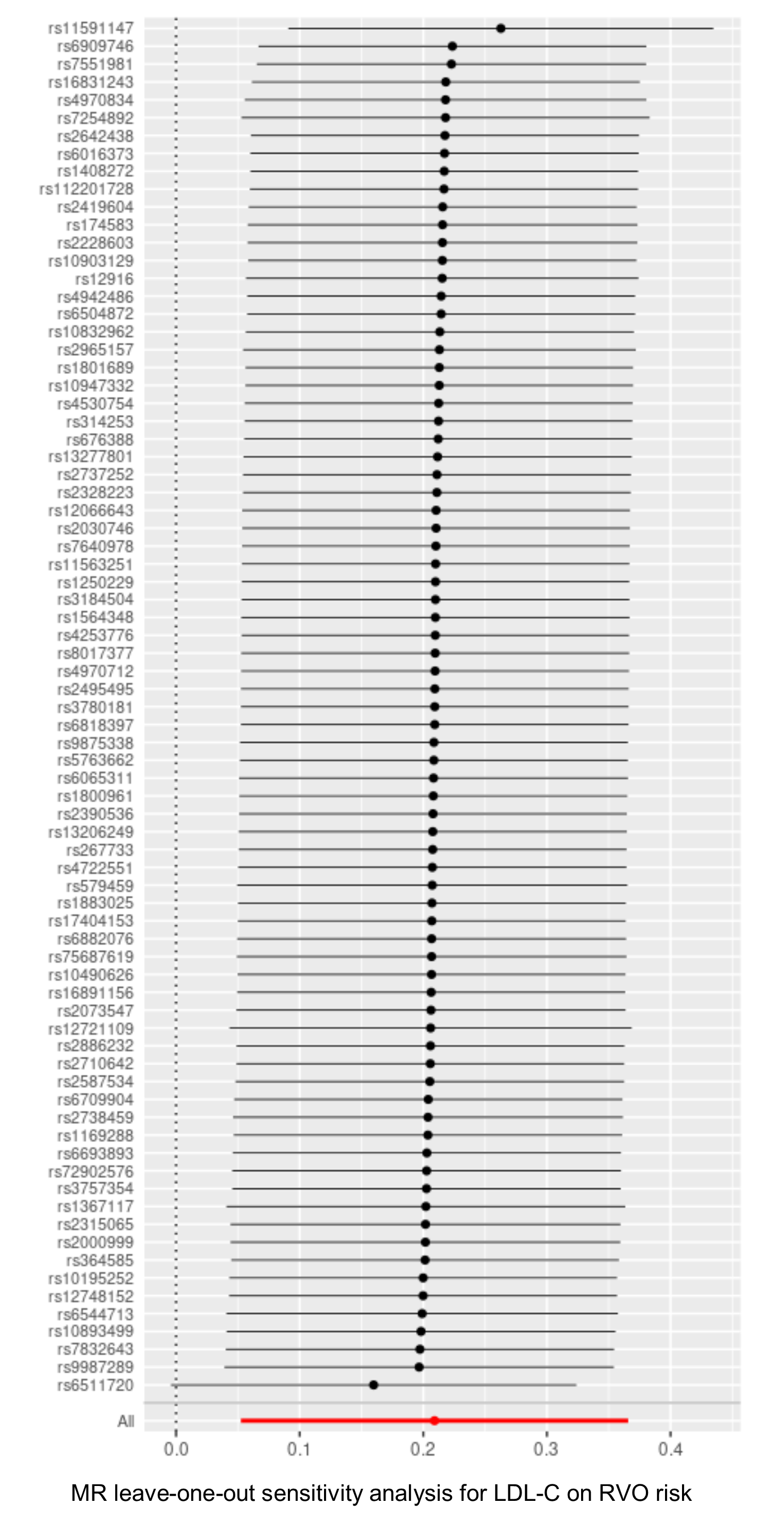


Figure 3:


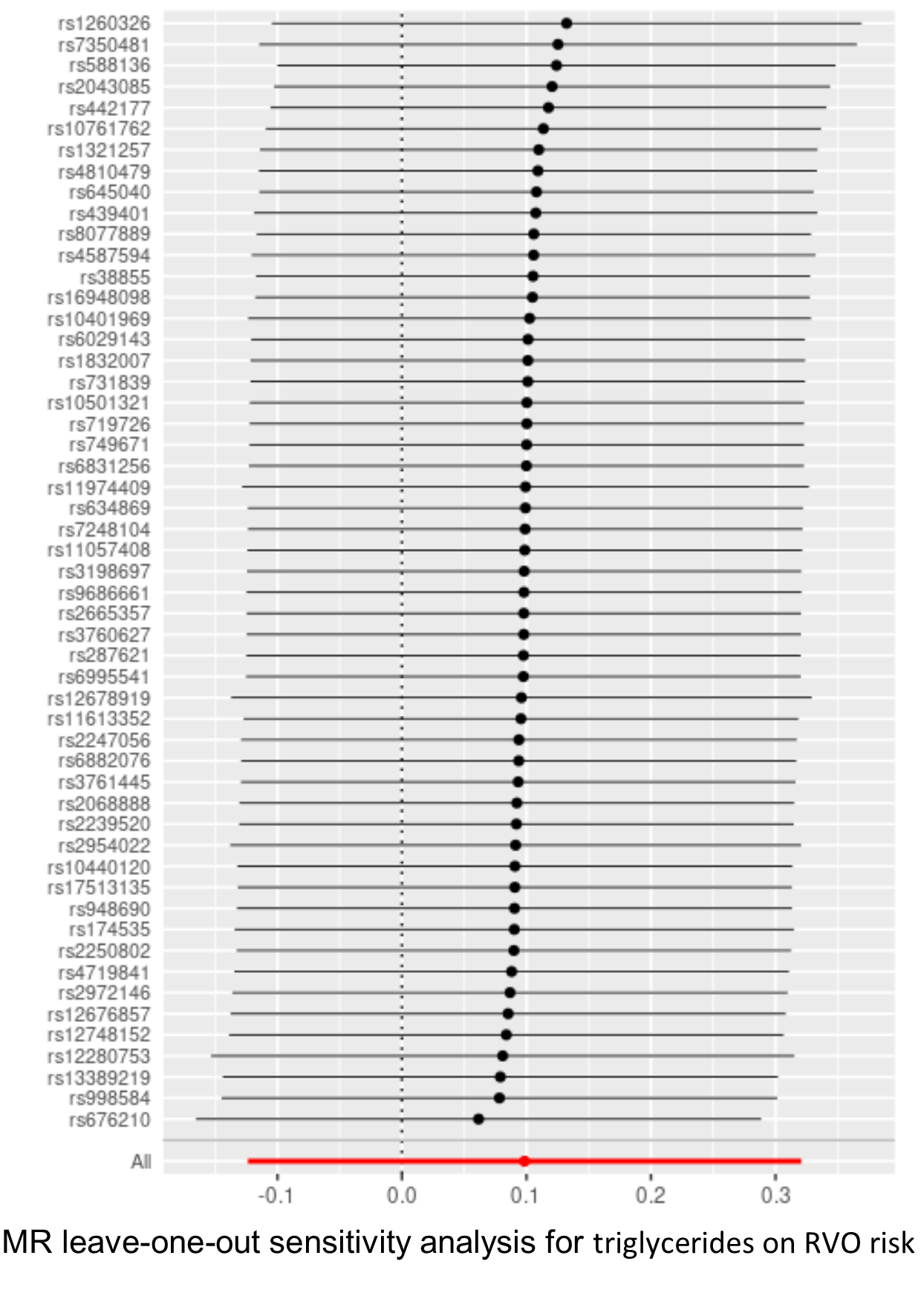


Figure 4:


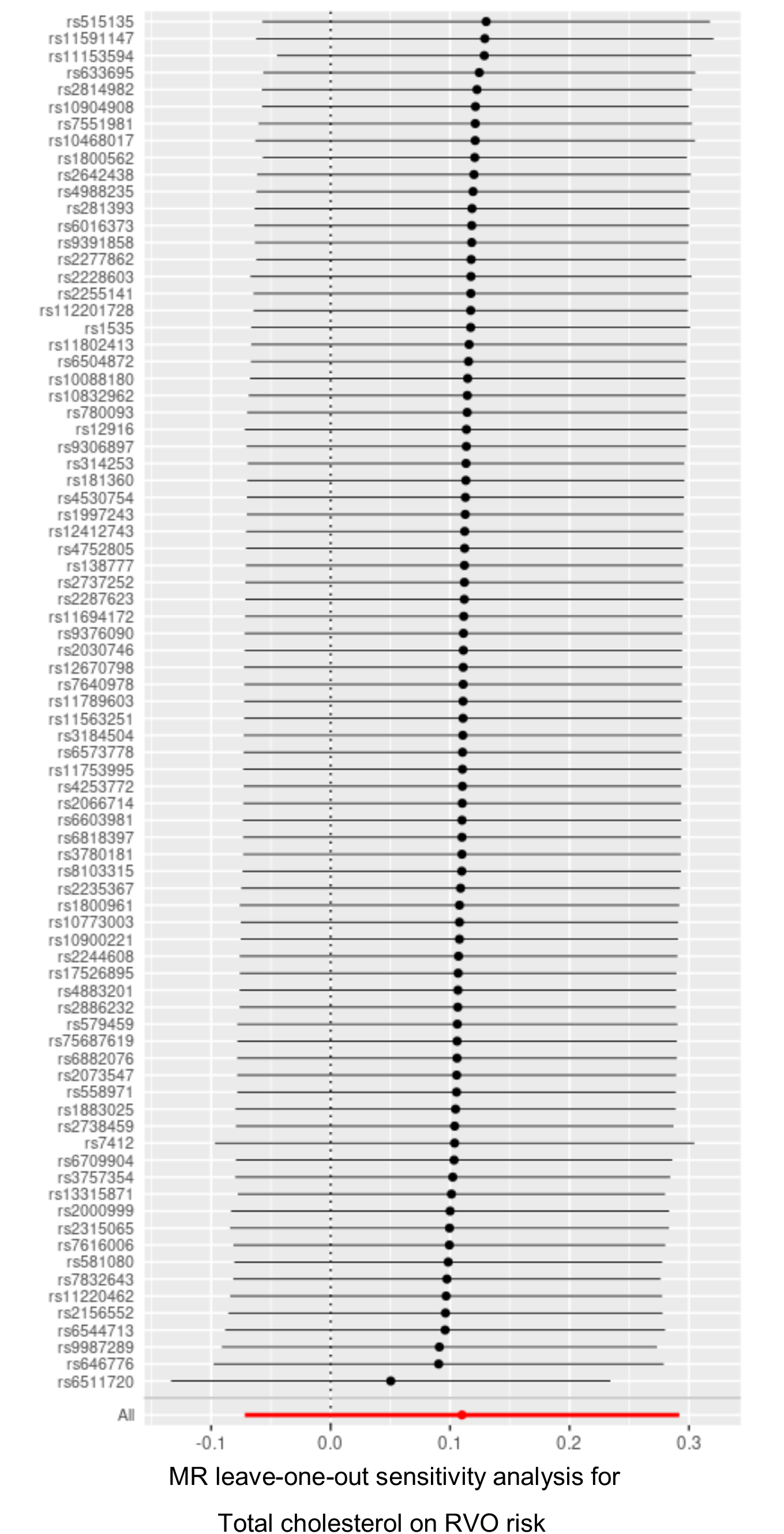


Figure 5：


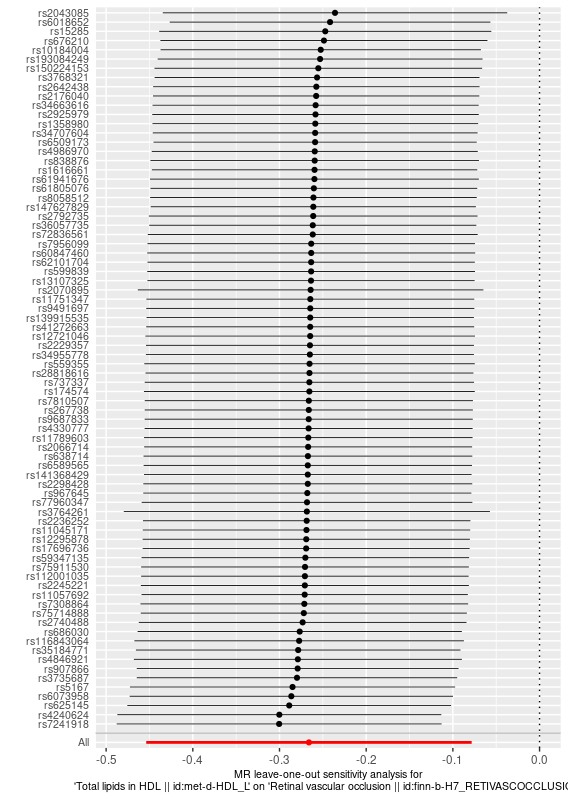


MR leave-one-out sensitivity analysis for HDL-C on RVO risk

Figure 6:


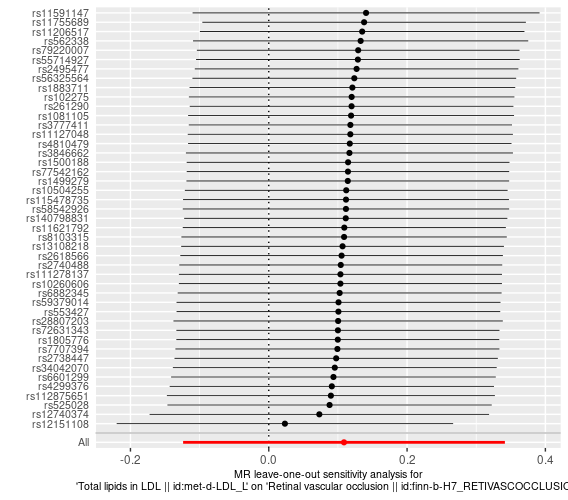


MR leave-one-out sensitivity analysis for LDL-C on RVO risk

Figure 7:


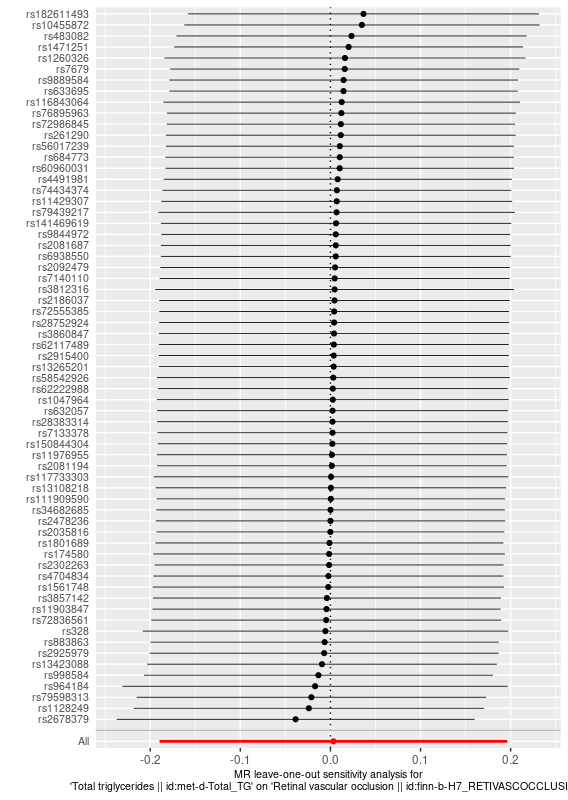


MR leave-one-out sensitivity analysis for triglycerides on RVO risk

Figure 8:


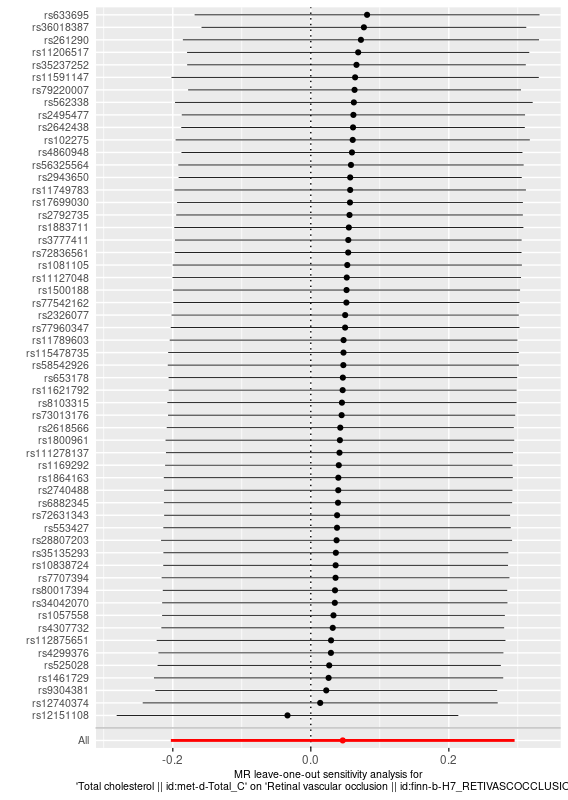


MR leave-one-out sensitivity analysis total cholesterol

on RVO risk
