# Supplementary material for: Plasma lipid levels and risk of retinal vascular occlusion: A genetic study using Mendelian randomization
Source: Front Endocrinol (Lausanne). 2022 Oct 10;13:954453. doi: 10.3389/fendo.2022.954453 (PMC9588969; doi:10.3389/fendo.2022.954453)
Supplement: Supplementary file 10 [file Table_2.docx]

Table 1: Information of Rucker'Q and Q-Q'. GLGC: Global Lipids Genetics Consortium. UKB: UK Biobank.

| Exposure |  | Cochrane's Q | | | Rucker's Q' | | | Q-Q' |  |  |
| --- | --- | --- | --- | --- | --- | --- | --- | --- | --- | --- |
|  | NSNP | Q | df | P value | Q | df | P value | Q-Q' | df | P value |
| GLGC |  |  |  |  |  |  |  |  |  |  |
| HDL-C | 86 | 90.256 | 85 | 0.328 | 89.549 | 84 | 0.319 | 0.707 | 1 | 0.4 |
| LDL-C | 77 | 74.604 | 76 | 0.524 | 74.349 | 75 | 0.499 | 0.255 | 1 | 0.613 |
| Triglycerides | 53 | 45.623 | 52 | 0.721 | 44.919 | 51 | 0.713 | 0.704 | 1 | 0.401 |
| Total cholesterol | 81 | 90.315 | 80 | 0.202 | 84.913 | 79 | 0.304 | 5.402 | 1 | 0.02 |
| UKB |  |  |  |  |  |  |  |  |  |  |
| HDL-C | 78 | 79.793 | 77 | 0.391 | 79.059 | 76 | 0.383 | 0.734 | 1 | 0.392 |
| LDL-C | 45 | 41.444 | 44 | 0.582 | 41.269 | 43 | 0.547 | 0.175 | 1 | 0.676 |
| Triglycerides | 65 | 63.561 | 64 | 0.492 | 63.489 | 63 | 0.459 | 0.072 | 1 | 0.788 |
| Total cholesterol | 57 | 65.242 | 56 | 0.186 | 65.167 | 55 | 0.164 | 0.075 | 1 | 0.784 |

Table 2: SNPs are overlapping with linkage disequilibrium with R^2^>0.8. GLGC: Global Lipids Genetics. UKB: UK Biobank. LD: linkage disequilibrium.

| HDL-C | | | LDL-C | | |
| --- | --- | --- | --- | --- | --- |
| GLGC | UKB | LD r^2^ | GLGC | UKB | LD r^2^ |
| rs11789603 | rs11789603 | 1 | rs11591147 | rs11591147 | 1 |
| rs13107325 | rs13107325 | 1 | rs13277801 | rs10504255 | 0.86 |
| rs2066714 | rs2066714 | 1 | rs174583 | rs102275 | 0.93 |
| rs2642438 | rs2642438 | 1 | rs2073547 | rs10260606 | 0.99 |
| rs4240624 | rs4240624 | 1 | rs75687619 | rs1081105 | 0.94 |
| rs676210 | rs676210 | 1 | rs579459 | rs115478735 | 0.82 |
| rs686030 | rs686030 | 1 | rs4530754 | rs1500188 | 0.96 |
| rs737337 | rs737337 | 1 | rs6511720 | rs12151108 | 0.97 |
| rs838876 | rs838876 | 1 | rs12916 | rs3846662 | 0.91 |
| rs998584 | rs1358980 | 0.83 | rs1883025 | rs2740488 | 0.96 |
| rs13702 | rs15285 | 0.99 | rs2000999 | rs34042070 | 0.94 |
| rs102275 | rs174574 | 0.96 | rs6544713 | rs4299376 | 0.97 |
| rs11065987 | rs17696736 | 0.96 | rs2587534 | rs553427 | 0.99 |
| rs1515110 | rs2176040 | 0.87 | rs6504872 | rs56325564 | 0.85 |
| rs3741414 | rs2229357 | 1 | rs1408272 | rs79220007 | 0.81 |
| rs181360 | rs2298428 | 0.89 | rs6882076 | rs6882345 | 1 |
| rs1883025 | rs2740488 | 0.96 | rs9987289 | rs6601299 | 0.84 |
| rs2293889 | rs2245221 | 0.84 |  |  |  |
| rs2250802 | rs2792735 | 0.96 |  |  |  |
| rs2925979 | rs2925979 | 1 |  |  |  |
| rs4846914 | rs4846921 | 0.95 |  |  |  |
| rs12740374 | rs599839 | 0.94 |  |  |  |
| rs499974 | rs559355 | 0.99 |  |  |  |
| rs1689797 | rs61805076 | 1 |  |  |  |
| rs4465830 | rs6073958 | 0.89 |  |  |  |
| rs4939883 | rs7241918 | 0.92 |  |  |  |
| rs2241210 | rs7308864 | 0.91 |  |  |  |
|  |  |  |  |  |  |
| Total cholesterol | | | Triglycerides | | |
| GLGC | UKB | LD r^2^ | GLGC | UKB | LD r^2^ |
| rs11591147 | rs11591147 | 1 | rs1260326 | rs1260326 | 1 |
| rs11789603 | rs11789603 | 1 | rs998584 | rs998584 | 1 |
| rs1800961 | rs1800961 | 1 | rs10401969 | rs58542926 | 0.95 |
| rs2642438 | rs2642438 | 1 | rs11057408 | rs7133378 | 0.88 |
| rs633695 | rs633695 | 1 | rs12678919 | rs328 | 0.98 |
| rs8103315 | rs8103315 | 1 | rs13389219 | rs1128249 | 1 |
| rs1535 | rs102275 | 0.91 | rs676210 | rs2678379 | 0.99 |
| rs75687619 | rs1081105 | 0.94 | rs634869 | rs632057 | 0.82 |
| rs780093 | rs11127048 | 0.89 | rs645040 | rs684773 | 0.93 |
| rs579459 | rs115478735 | 0.82 | rs6882076 | rs4704834 | 0.87 |
| rs6573778 | rs11621792 | 0.92 |  |  |  |
| rs12916 | rs11749783 | 0.90 |  |  |  |
| rs2244608 | rs1169292 | 0.88 |  |  |  |
| rs646776 | rs12740374 | 1 |  |  |  |
| rs6511720 | rs12151108 | 0.97 |  |  |  |
| rs9987289 | rs1461729 | 0.82 |  |  |  |
| rs4530754 | rs1500188 | 0.96 |  |  |  |
| rs10468017 | rs261290 | 0.83 |  |  |  |
| rs1883025 | rs2740488 | 0.96 |  |  |  |
| rs2255141 | rs2792735 | 0.96 |  |  |  |
| rs2000999 | rs34042070 | 0.94 |  |  |  |
| rs6544713 | rs4299376 | 0.97 |  |  |  |
| rs3184504 | rs653178 | 0.94 |  |  |  |
| rs515135 | rs562338 | 0.97 |  |  |  |
| rs558971 | rs553427 | 0.98 |  |  |  |
| rs6504872 | rs56325564 | 0.85 |  |  |  |
| rs6882076 | rs6882345 | 1 |  |  |  |
| rs1800562 | rs79220007 | 1 |  |  |  |
| rs2156552 | rs9304381 | 0.92 |  |  |  |
